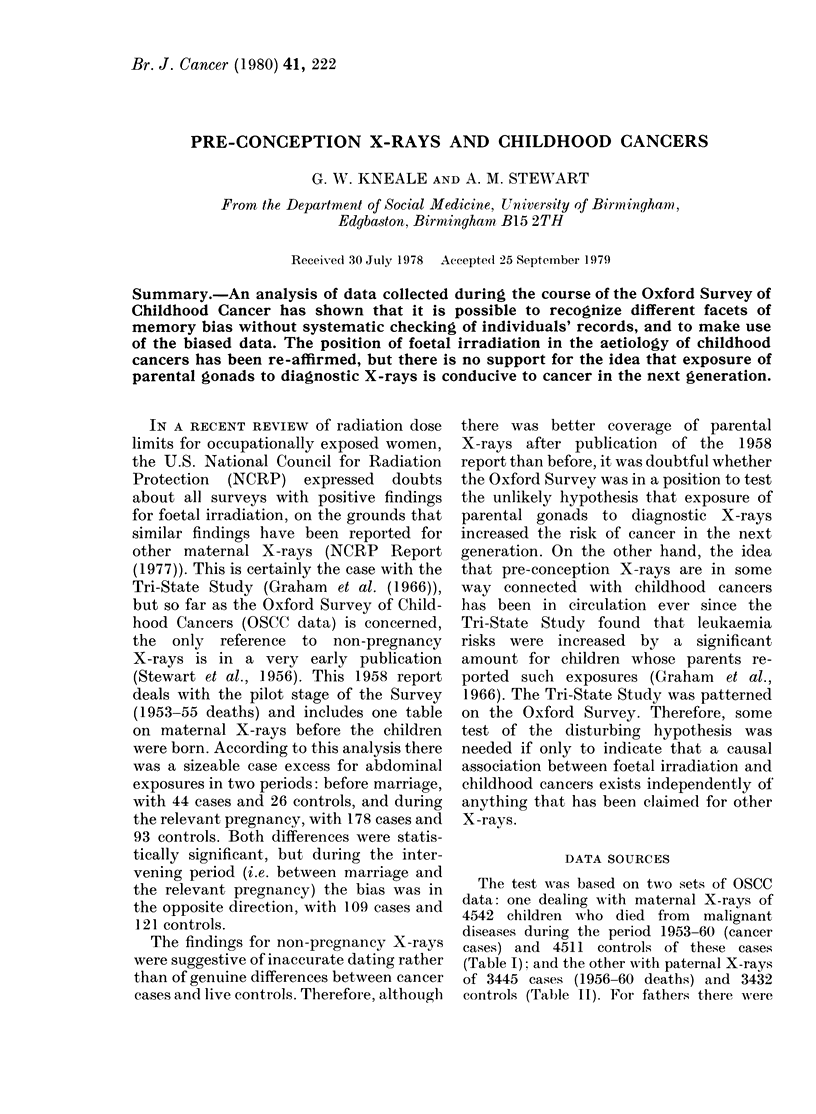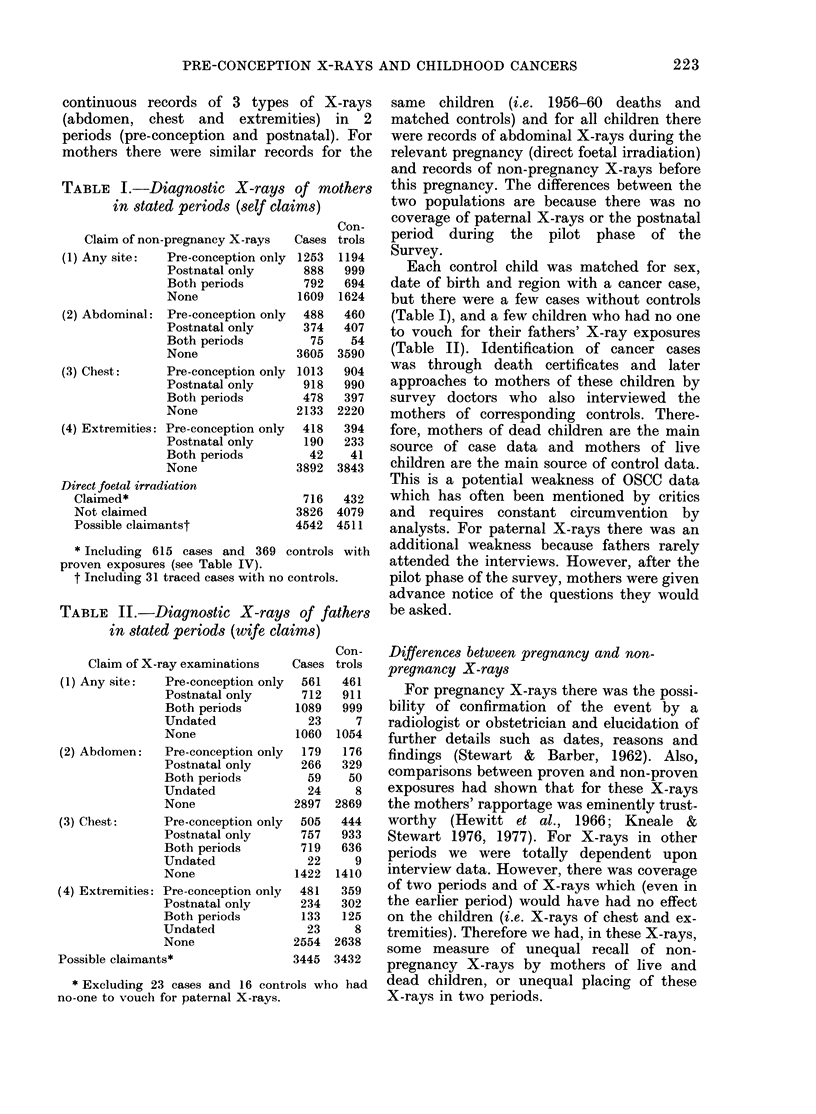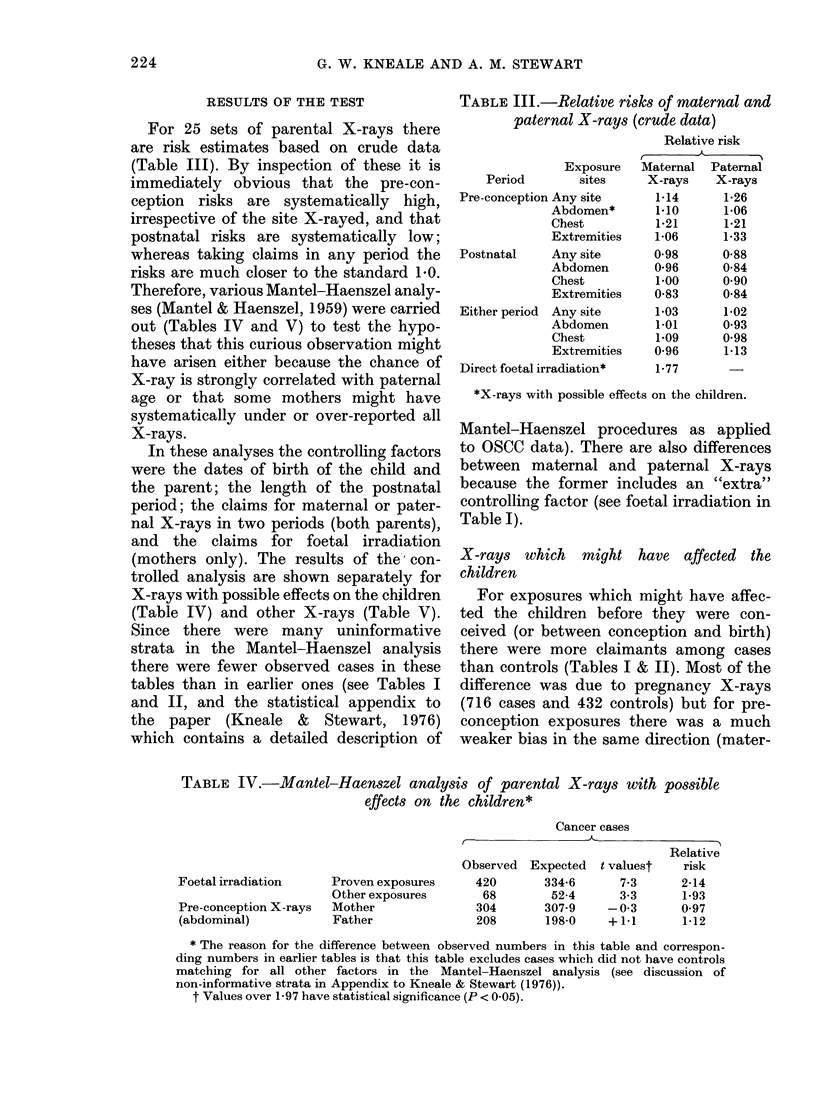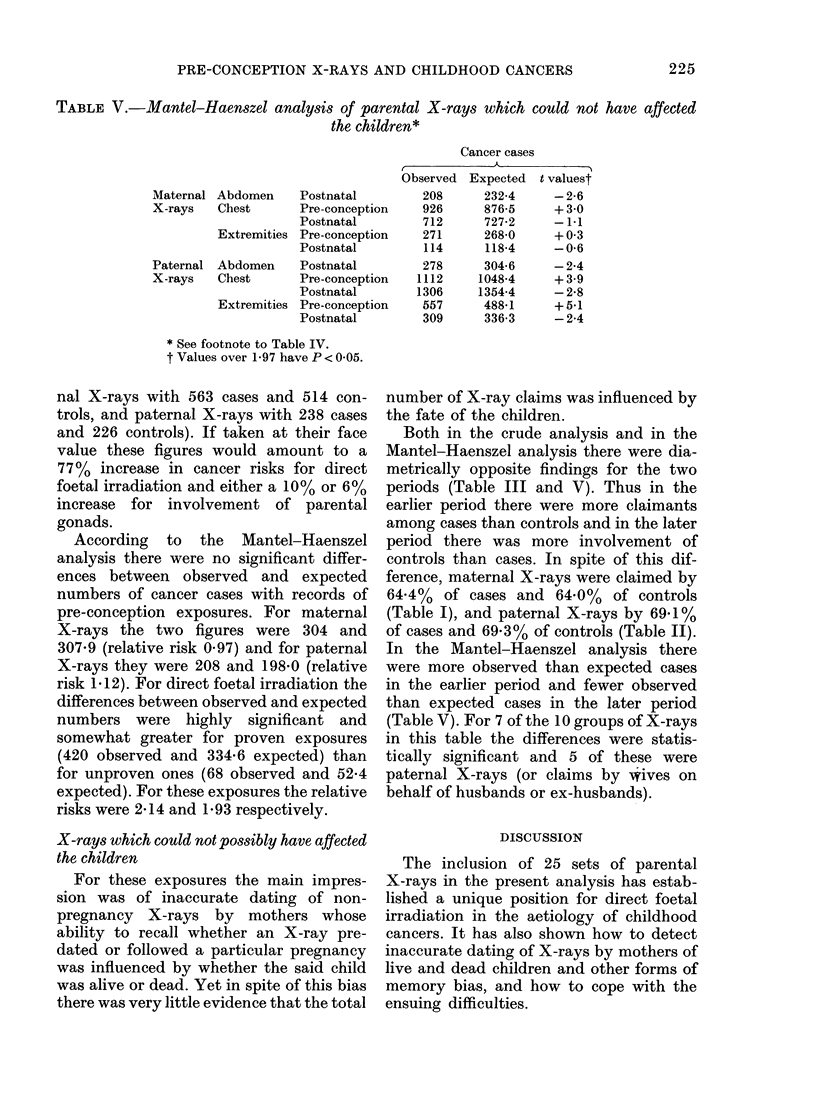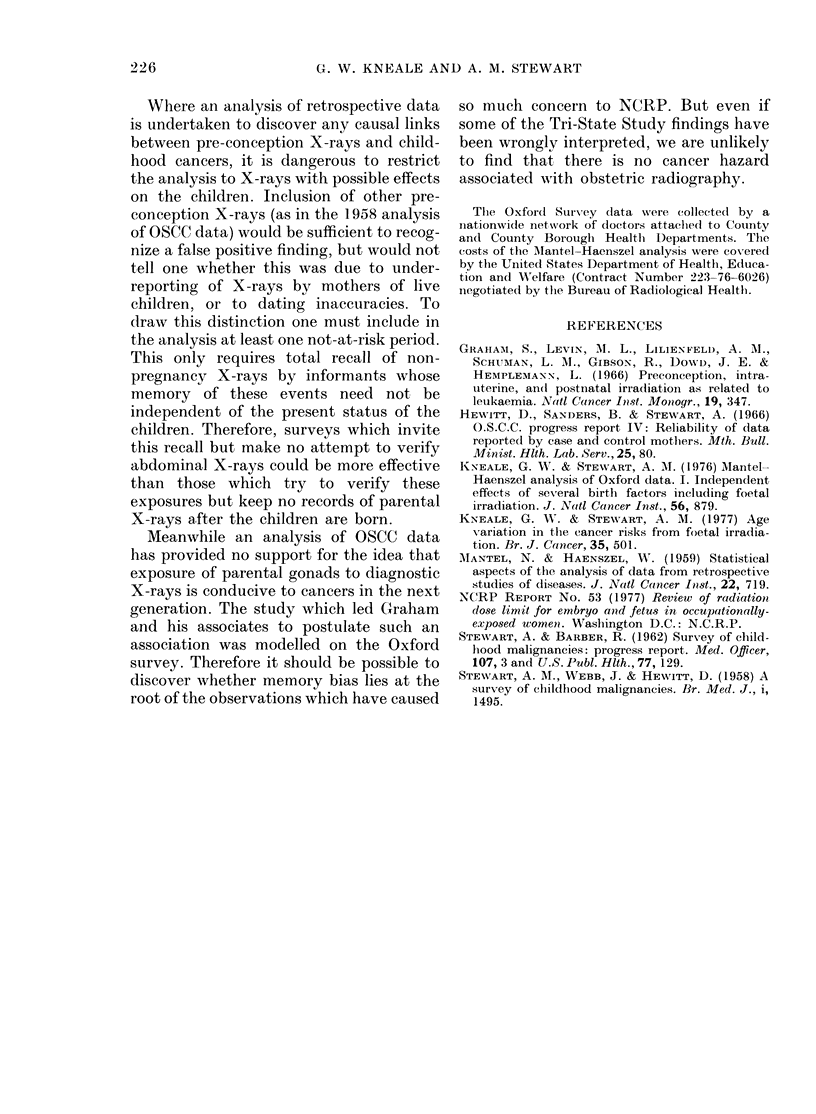# Pre-conception X-rays and childhood cancers.

**DOI:** 10.1038/bjc.1980.33

**Published:** 1980-02

**Authors:** G. W. Kneale, A. M. Stewart

## Abstract

An analysis of data collected during the course of the Oxford Survey of Childhood Cancer has shown that it is possible to recognize different facets of memory bias without systematic checking of individuals' records, and to make use of the biased data. The position of foetal irradiation in the aetiology of childhood cancers has been re-affirmed, but there is no support for the idea that exposure of parental gonads to diagnostic X-rays is conducive to cancer in the next generation.


*Br. J. Cancer* (1980) **41**, 222

### PRE-CONCEPTION X-RAYS AND CHILDHOOD CANCERS

G. W. KNEALE AND A. M. STEWART

*From the Department of Social Medicine, University of Birmingham, Edgbaston, Birmingham B15 2TH*

Received 30 July 1978 Accepted 25 September 1979

**Summary.—An analysis of data collected during the course of the Oxford Survey of Childhood Cancer has shown that it is possible to recognize different facets of memory bias without systematic checking of individuals' records, and to make use of the biased data. The position of foetal irradiation in the aetiology of childhood cancers has been re-affirmed, but there is no support for the idea that exposure of parental gonads to diagnostic X-rays is conducive to cancer in the next generation.**

IN A RECENT REVIEW of radiation dose limits for occupationally exposed women, the U.S. National Council for Radiation Protection (NCRP) expressed doubts about all surveys with positive findings for foetal irradiation, on the grounds that similar findings have been reported for other maternal X-rays (NCRP Report (1977)). This is certainly the case with the Tri-State Study (Graham *et al.* (1966)), but so far as the Oxford Survey of Childhood Cancers (OSCC data) is concerned, the only reference to non-pregnancy X-rays is in a very early publication (Stewart *et al.*, 1956). This 1958 report deals with the pilot stage of the Survey (1953–55 deaths) and includes one table on maternal X-rays before the children were born. According to this analysis there was a sizeable case excess for abdominal exposures in two periods: before marriage, with 44 cases and 26 controls, and during the relevant pregnancy, with 178 cases and 93 controls. Both differences were statistically significant, but during the intervening period (*i.e.* between marriage and the relevant pregnancy) the bias was in the opposite direction, with 109 cases and 121 controls.

The findings for non-pregnancy X-rays were suggestive of inaccurate dating rather than of genuine differences between cancer cases and live controls. Therefore, although there was better coverage of parental X-rays after publication of the 1958 report than before, it was doubtful whether the Oxford Survey was in a position to test the unlikely hypothesis that exposure of parental gonads to diagnostic X-rays increased the risk of cancer in the next generation. On the other hand, the idea that pre-conception X-rays are in some way connected with childhood cancers has been in circulation ever since the Tri-State Study found that leukaemia risks were increased by a significant amount for children whose parents reported such exposures (Graham *et al.*, 1966). The Tri-State Study was patterned on the Oxford Survey. Therefore, some test of the disturbing hypothesis was needed if only to indicate that a causal association between foetal irradiation and childhood cancers exists independently of anything that has been claimed for other X-rays.

### DATA SOURCES

The test was based on two sets of OSCC data: one dealing with maternal X-rays of 4542 children who died from malignant diseases during the period 1953–60 (cancer cases) and 4511 controls of these cases (Table I); and the other with paternal X-rays of 3445 cases (1956–60 deaths) and 3432 controls (Table II). For fathers there were continuous records of 3 types of X-rays (abdomen, chest and extremities) in 2 periods (pre-conception and postnatal). For mothers there were similar records for the same children (*i.e.* 1956–60 deaths and matched controls) and for all children there were records of abdominal X-rays during the relevant pregnancy (direct foetal irradiation) and records of non-pregnancy X-rays before this pregnancy. The differences between the two populations are because there was no coverage of paternal X-rays or the postnatal period during the pilot phase of the Survey.

TABLE I.—*Diagnostic X-rays of mothers in stated periods (self claims)*

| Claim of non-pregnancy X-rays | | Cases | Controls |
|---|---|---|---|
| (1) Any site: | Pre-conception only | 1253 | 1194 |
| | Postnatal only | 888 | 999 |
| | Both periods | 792 | 694 |
| | None | 1609 | 1624 |
| (2) Abdominal: | Pre-conception only | 488 | 460 |
| | Postnatal only | 374 | 407 |
| | Both periods | 75 | 54 |
| | None | 3605 | 3590 |
| (3) Chest: | Pre-conception only | 1013 | 904 |
| | Postnatal only | 918 | 990 |
| | Both periods | 478 | 397 |
| | None | 2133 | 2220 |
| (4) Extremities: | Pre-conception only | 418 | 394 |
| | Postnatal only | 190 | 233 |
| | Both periods | 42 | 41 |
| | None | 3892 | 3843 |
| *Direct foetal irradiation* | | | |
| Claimed* | | 716 | 432 |
| Not claimed | | 3826 | 4079 |
| Possible claimants† | | 4542 | 4511 |

* Including 615 cases and 369 controls with proven exposures (see Table IV).

† Including 31 traced cases with no controls.

TABLE II.—*Diagnostic X-rays of fathers in stated periods (wife claims)*

| Claim of X-ray examinations | | Cases | Controls |
|---|---|---|---|
| (1) Any site: | Pre-conception only | 561 | 461 |
| | Postnatal only | 712 | 911 |
| | Both periods | 1089 | 999 |
| | Undated | 23 | 7 |
| | None | 1060 | 1054 |
| (2) Abdomen: | Pre-conception only | 179 | 176 |
| | Postnatal only | 266 | 329 |
| | Both periods | 59 | 50 |
| | Undated | 24 | 8 |
| | None | 2897 | 2869 |
| (3) Chest: | Pre-conception only | 505 | 444 |
| | Postnatal only | 757 | 933 |
| | Both periods | 719 | 636 |
| | Undated | 22 | 9 |
| | None | 1422 | 1410 |
| (4) Extremities: | Pre-conception only | 481 | 359 |
| | Postnatal only | 234 | 302 |
| | Both periods | 133 | 125 |
| | Undated | 23 | 8 |
| | None | 2554 | 2638 |
| Possible claimants* | | 3445 | 3432 |

* Excluding 23 cases and 16 controls who had no-one to vouch for paternal X-rays.

Each control child was matched for sex, date of birth and region with a cancer case, but there were a few cases without controls (Table I), and a few children who had no one to vouch for their fathers' X-ray exposures (Table II). Identification of cancer cases was through death certificates and later approaches to mothers of these children by survey doctors who also interviewed the mothers of corresponding controls. Therefore, mothers of dead children are the main source of case data and mothers of live children are the main source of control data. This is a potential weakness of OSCC data which has often been mentioned by critics and requires constant circumvention by analysts. For paternal X-rays there was an additional weakness because fathers rarely attended the interviews. However, after the pilot phase of the survey, mothers were given advance notice of the questions they would be asked.

*Differences between pregnancy and non-pregnancy X-rays*

For pregnancy X-rays there was the possibility of confirmation of the event by a radiologist or obstetrician and elucidation of further details such as dates, reasons and findings (Stewart & Barber, 1962). Also, comparisons between proven and non-proven exposures had shown that for these X-rays the mothers' rapportage was eminently trustworthy (Hewitt *et al.*, 1966; Kneale & Stewart 1976, 1977). For X-rays in other periods we were totally dependent upon interview data. However, there was coverage of two periods and of X-rays which (even in the earlier period) would have had no effect on the children (*i.e.* X-rays of chest and extremities). Therefore we had, in these X-rays, some measure of unequal recall of non-pregnancy X-rays by mothers of live and dead children, or unequal placing of these X-rays in two periods.

RESULTS OF THE TEST

For 25 sets of parental X-rays there are risk estimates based on crude data (Table III). By inspection of these it is immediately obvious that the pre-conception risks are systematically high, irrespective of the site X-rayed, and that postnatal risks are systematically low; whereas taking claims in any period the risks are much closer to the standard 1·0. Therefore, various Mantel–Haenszel analyses (Mantel & Haenszel, 1959) were carried out (Tables IV and V) to test the hypotheses that this curious observation might have arisen either because the chance of X-ray is strongly correlated with paternal age or that some mothers might have systematically under or over-reported all X-rays.

In these analyses the controlling factors were the dates of birth of the child and the parent; the length of the postnatal period; the claims for maternal or paternal X-rays in two periods (both parents), and the claims for foetal irradiation (mothers only). The results of the controlled analysis are shown separately for X-rays with possible effects on the children (Table IV) and other X-rays (Table V). Since there were many uninformative strata in the Mantel–Haenszel analysis there were fewer observed cases in these tables than in earlier ones (see Tables I and II, and the statistical appendix to the paper (Kneale & Stewart, 1976) which contains a detailed description of Mantel–Haenszel procedures as applied to OSCC data). There are also differences between maternal and paternal X-rays because the former includes an "extra" controlling factor (see foetal irradiation in Table I).

TABLE III.—*Relative risks of maternal and paternal X-rays (crude data)*

| Period | Exposure sites | Relative risk: Maternal X-rays | Relative risk: Paternal X-rays |
|---|---|---|---|
| Pre-conception | Any site | 1·14 | 1·26 |
| | Abdomen* | 1·10 | 1·06 |
| | Chest | 1·21 | 1·21 |
| | Extremities | 1·06 | 1·33 |
| Postnatal | Any site | 0·98 | 0·88 |
| | Abdomen | 0·96 | 0·84 |
| | Chest | 1·00 | 0·90 |
| | Extremities | 0·83 | 0·84 |
| Either period | Any site | 1·03 | 1·02 |
| | Abdomen | 1·01 | 0·93 |
| | Chest | 1·09 | 0·98 |
| | Extremities | 0·96 | 1·13 |
| Direct foetal irradiation* | | 1·77 | — |

*X-rays with possible effects on the children.

*X-rays which might have affected the children*

For exposures which might have affected the children before they were conceived (or between conception and birth) there were more claimants among cases than controls (Tables I & II). Most of the difference was due to pregnancy X-rays (716 cases and 432 controls) but for pre-conception exposures there was a much weaker bias in the same direction (mater-

TABLE IV.—*Mantel–Haenszel analysis of parental X-rays with possible effects on the children**

| | | Cancer cases: Observed | Expected | t values† | Relative risk |
|---|---|---|---|---|---|
| Foetal irradiation | Proven exposures | 420 | 334·6 | 7·3 | 2·14 |
| | Other exposures | 68 | 52·4 | 3·3 | 1·93 |
| Pre-conception X-rays | Mother | 304 | 307·9 | −0·3 | 0·97 |
| (abdominal) | Father | 208 | 198·0 | +1·1 | 1·12 |

* The reason for the difference between observed numbers in this table and corresponding numbers in earlier tables is that this table excludes cases which did not have controls matching for all other factors in the Mantel–Haenszel analysis (see discussion of non-informative strata in Appendix to Kneale & Stewart (1976)).

† Values over 1·97 have statistical significance ($P < 0{\cdot}05$).

TABLE V.—*Mantel–Haenszel analysis of parental X-rays which could not have affected the children**

| | | | Cancer cases | | |
|---|---|---|---|---|---|
| | | | Observed | Expected | $t$ values† |
| Maternal X-rays | Abdomen | Postnatal | 208 | 232·4 | −2·6 |
| | Chest | Pre-conception | 926 | 876·5 | +3·0 |
| | | Postnatal | 712 | 727·2 | −1·1 |
| | Extremities | Pre-conception | 271 | 268·0 | +0·3 |
| | | Postnatal | 114 | 118·4 | −0·6 |
| Paternal X-rays | Abdomen | Postnatal | 278 | 304·6 | −2·4 |
| | Chest | Pre-conception | 1112 | 1048·4 | +3·9 |
| | | Postnatal | 1306 | 1354·4 | −2·8 |
| | Extremities | Pre-conception | 557 | 488·1 | +5·1 |
| | | Postnatal | 309 | 336·3 | −2·4 |

* See footnote to Table IV.

† Values over 1·97 have $P < 0{\cdot}05$.

nal X-rays with 563 cases and 514 controls, and paternal X-rays with 238 cases and 226 controls). If taken at their face value these figures would amount to a 77% increase in cancer risks for direct foetal irradiation and either a 10% or 6% increase for involvement of parental gonads.

According to the Mantel–Haenszel analysis there were no significant differences between observed and expected numbers of cancer cases with records of pre-conception exposures. For maternal X-rays the two figures were 304 and 307·9 (relative risk 0·97) and for paternal X-rays they were 208 and 198·0 (relative risk 1·12). For direct foetal irradiation the differences between observed and expected numbers were highly significant and somewhat greater for proven exposures (420 observed and 334·6 expected) than for unproven ones (68 observed and 52·4 expected). For these exposures the relative risks were 2·14 and 1·93 respectively.

*X-rays which could not possibly have affected the children*

For these exposures the main impression was of inaccurate dating of non-pregnancy X-rays by mothers whose ability to recall whether an X-ray predated or followed a particular pregnancy was influenced by whether the said child was alive or dead. Yet in spite of this bias there was very little evidence that the total number of X-ray claims was influenced by the fate of the children.

Both in the crude analysis and in the Mantel–Haenszel analysis there were diametrically opposite findings for the two periods (Table III and V). Thus in the earlier period there were more claimants among cases than controls and in the later period there was more involvement of controls than cases. In spite of this difference, maternal X-rays were claimed by 64·4% of cases and 64·0% of controls (Table I), and paternal X-rays by 69·1% of cases and 69·3% of controls (Table II). In the Mantel–Haenszel analysis there were more observed than expected cases in the earlier period and fewer observed than expected cases in the later period (Table V). For 7 of the 10 groups of X-rays in this table the differences were statistically significant and 5 of these were paternal X-rays (or claims by wives on behalf of husbands or ex-husbands).

### DISCUSSION

The inclusion of 25 sets of parental X-rays in the present analysis has established a unique position for direct foetal irradiation in the aetiology of childhood cancers. It has also shown how to detect inaccurate dating of X-rays by mothers of live and dead children and other forms of memory bias, and how to cope with the ensuing difficulties.

Where an analysis of retrospective data is undertaken to discover any causal links between pre-conception X-rays and childhood cancers, it is dangerous to restrict the analysis to X-rays with possible effects on the children. Inclusion of other pre-conception X-rays (as in the 1958 analysis of OSCC data) would be sufficient to recognize a false positive finding, but would not tell one whether this was due to under-reporting of X-rays by mothers of live children, or to dating inaccuracies. To draw this distinction one must include in the analysis at least one not-at-risk period. This only requires total recall of non-pregnancy X-rays by informants whose memory of these events need not be independent of the present status of the children. Therefore, surveys which invite this recall but make no attempt to verify abdominal X-rays could be more effective than those which try to verify these exposures but keep no records of parental X-rays after the children are born.

Meanwhile an analysis of OSCC data has provided no support for the idea that exposure of parental gonads to diagnostic X-rays is conducive to cancers in the next generation. The study which led Graham and his associates to postulate such an association was modelled on the Oxford survey. Therefore it should be possible to discover whether memory bias lies at the root of the observations which have caused so much concern to NCRP. But even if some of the Tri-State Study findings have been wrongly interpreted, we are unlikely to find that there is no cancer hazard associated with obstetric radiography.

The Oxford Survey data were collected by a nationwide network of doctors attached to County and County Borough Health Departments. The costs of the Mantel–Haenszel analysis were covered by the United States Department of Health, Education and Welfare (Contract Number 223–76–6026) negotiated by the Bureau of Radiological Health.

### REFERENCES

Graham, S., Levin, M. L., Lilienfeld, A. M., Schuman, L. M., Gibson, R., Dowd, J. E. & Hemplemann, L. (1966) Preconception, intrauterine, and postnatal irradiation as related to leukaemia. *Natl Cancer Inst. Monogr.*, **19**, 347.

Hewitt, D., Sanders, B. & Stewart, A. (1966) O.S.C.C. progress report IV: Reliability of data reported by case and control mothers. *Mth. Bull. Minist. Hlth. Lab. Serv.*, **25**, 80.

Kneale, G. W. & Stewart, A. M. (1976) Mantel–Haenszel analysis of Oxford data. I. Independent effects of several birth factors including foetal irradiation. *J. Natl Cancer Inst.*, **56**, 879.

Kneale, G. W. & Stewart, A. M. (1977) Age variation in the cancer risks from foetal irradiation. *Br. J. Cancer*, **35**, 501.

Mantel, N. & Haenszel, W. (1959) Statistical aspects of the analysis of data from retrospective studies of diseases. *J. Natl Cancer Inst.*, **22**, 719.

NCRP Report No. 53 (1977) *Review of radiation dose limit for embryo and fetus in occupationally-exposed women.* Washington D.C.: N.C.R.P.

Stewart, A. & Barber, R. (1962) Survey of childhood malignancies: progress report. *Med. Officer*, **107**, 3 and *U.S. Publ. Hlth.*, **77**, 129.

Stewart, A. M., Webb, J. & Hewitt, D. (1958) A survey of childhood malignancies. *Br. Med. J.*, **i**, 1495.